# Time-and-motion tool for the assessment of working time in tuberculosis laboratories: a multicentre study

**DOI:** 10.5588/ijtld.17.0564

**Published:** 2018-04-01

**Authors:** V. Mathys, E. Roycroft, P. Raftery, R. Groenheit, D. B. Folkvardsen, D. Homorodean, E. Vasiliauskiene, L. Vasiliauskaite, C. Kodmon, M. J. van der Werf, F. Drobniewski, V. Nikolayevskyy

**Affiliations:** * Scientific Institute of Public Health (WIV-ISP), Brussels, Belgium; † Irish Mycobacteria Reference Laboratory, St James' Hospital, Dublin, Ireland; ‡ Public Health Agency of Sweden, Stockholm, Sweden; § International Reference Laboratory of Mycobacteriology, Statens Serum Institut, Copenhagen, Denmark; ¶ Clinical Hospital of Pneumology, Cluj-Napoca, Romania; # Centre of Laboratory Medicine, Tuberculosis Laboratory, Vilnius University Hospital Santaros Klinikos, Vilnius; ** Institute of Biomedical Sciences, Department of Physiology, Biochemistry, Microbiology and Laboratory Medicine, Faculty of Medicine, Vilnius University, Vilnius, Lithuania; †† European Centre for Disease Prevention and Control, Stockholm, Sweden; ‡‡ Imperial College, London; §§ Public Health England, National Mycobacterium Reference Service South, London, UK

**Keywords:** workload, hands-on time, laboratory diagnosis

## Abstract

**SETTING:**

Implementation of novel diagnostic assays in tuberculosis (TB) laboratory diagnosis requires effective management of time and resources.

**OBJECTIVE:**

To further develop and assess at multiple centres a time-and-motion (T&M) tool as an objective means for recording the actual time spent on running laboratory assays.

**DESIGN:**

Multicentre prospective study conducted in six European Union (EU) reference TB laboratories.

**RESULTS:**

A total of 1060 specimens were tested using four laboratory assays. The number of specimens per batch varied from one to 60; a total of 64 recordings were performed. Theoretical hands-on times per specimen (TTPS) in h:min:s for Xpert^®^ MTB/RIF, mycobacterial interspersed repetitive unit-variable number of tandem repeats genotyping, Ziehl-Neelsen staining and manual fluorescence microscopy were respectively 00:33:02 ± 00:12:32, 00:13:34 ± 00:03:11, 00:09:54 ± 00:00:53 and 00:06:23 ± 00:01:36. Variations between laboratories were predominantly linked to the time spent on reporting and administrative procedures. Processing specimens in batches could help save time in highly automated assays (e.g., line-probe) (TTPS 00:14:00 vs. 00:09:45 for batches comprising 7 and 31 specimens, respectively).

**CONCLUSIONS:**

The T&M tool can be considered a universal and objective methodology contributing to workload assessment in TB diagnostic laboratories. Comparison of workload between laboratories could help laboratory managers justify their resource and personnel needs for the implementation of novel, time-saving, cost-effective technologies, as well as identify areas for improvement.

## Abstract (FR)

**CONTEXTE:**

La mise en œuvre de nouveaux tests de diagnostic de la tuberculose (TB) en laboratoire requiert une gestion efficace du temps et des ressources.

**OBJECTIF:**

Développer et évaluer dans des multiples centres un outil d'étude des temps et mouvements (T&M) comme moyen d'enregistrer le temps réellement consacré aux tests de laboratoire.

**SCHÉMA:**

Etude prospective multicentrique réalisée dans six laboratoires de référence de la TB différents de l'Union Européenne.

**RÉSULTATS:**

Un total de 1060 échantillons ont été testés avec quatre tests de laboratoire. Le nombre d'échantillons par lot a varié de un à 60, avec un total de 64 enregistrements réalisés. Le temps théorique de manipulation par échantillon (TTPS) pour l'Xpert® MTB/RIF, le génotypage par la méthode des unités répétitives dispersées sur le génome mycobactériennombre variable de répétitions en tandem, la coloration Ziehl-Neelsen et la microscopie de fluorescence à evaluation manuelle a été de 00:33:02 ± 00:12:32 (h:min:s), 00:13:34 ± 00:03:11, 00:09:54 ± 00:00:53 et 00:06:23 ± 00:01:36, respectivement. Les variations entre laboratoires ont été surtout liées au temps consacré aux rapports et aux procédures administratives. Le traitement des échantillons dans les lots peut contribuer à gagner du temps pour les tests hautement automatisés (par exemple, les sondes en ligne) (TTPS 00:14:00 contre 00:09:45 pour les lots comprenant 7 et 31 échantillons, respectivement).

**CONCLUSION:**

L'outil T&M peut être considéré comme une méthode universelle et objective contribuant à l'évaluation de la charge de travail des laboratoires de diagnostic de la TB. La comparaison de la charge de travail entre laboratoires pourrait aider les gestionnaires des laboratoires à justifier leurs besoins en ressources et en personnel pour la mise en œuvre de techniques nouvelles,épargnant du temps et rentables, ainsi qu'à identifier les domaines à améliorer.

## Abstract (ES)

**MARCO DE REFERENCIA:**

La introducción de nuevas pruebas en los laboratorios de diagnóstico de la tuberculosis (TB) exige una gestión eficaz del tiempo y de los recursos.

**OBJETIVO:**

Ampliar el desarrollo y realizar una evaluación multicéntrica de un instrumento de análisis de tiempos y movimientos (T&M), como un medio objetivo de registrar el tiempo real empleado en la realización de pruebas de laboratorio.

**MÉTODO:**

Se llevó a cabo un estudio prospectivo multicéntrico en seis laboratorios de referencia de TB de la Unión Europea.

**RESULTADOS:**

Se analizaron 1060 muestras mediante cuatro pruebas de laboratorio. El número de muestras por lote osciló entre uno y 60 y se realizaron 64 registros. El tiempo teórico invertido por muestra con la prueba Xpert^®^ MTB/RIF fue 00:33:02 ± 00:12:32 (h:min:s), con la genotipificación de unidades micobacterianas incercaladas repetidas-numéro variable de repeticiones en tándem fue 00:13:34 ± 00:03:11, con la coloración de Ziehl-Neelsen 00:09:54 ± 00:00:53 y con el examen manual por microscopia fluorescente fue 00:06:23 ± 00:01:36. Las variaciones entre los laboratorios dependieron en su mayor parte del tiempo dedicado a la notificación y los procedimientos administrativos. El procesamiento de las muestras por lotes puede ayudar a ahorrar tiempo en los análisis muy automatizados (como la hibridación con sondas en tiras, 00:14:00 contra 00:09:45 en lotes de siete a 31 muestras, respectivamente).

**CONCLUSIÓN:**

El instrumento de análisis T&M se puede considerar como un método objetivo de evaluación de la carga de trabajo en los laboratorios de diagnóstico de la TB. Comparar la carga de trabajo entre los laboratorios podría ayudar a los directores de laboratorio a justificar sus necesidades de recursos y personal, cuando se planea la introducción de nuevas tecnologías rentables, que ahorran tiempo y también a definir las esferas que precisan mejoramiento.

TUBERCULOSIS (TB) is the most deadly communicable disease worldwide. In 2015, about 10.4 million people developed TB and 1.8 million died from it.^1^ TB control is further complicated by the spread of multidrug-resistant TB (MDR-TB), which requires lengthier treatment than for susceptible TB, is much more expensive to treat and frequently results in unsuccessful treatment outcomes. In the European Union (EU), less than 50% of MDR-TB cases are treated successfully.^2^

Timely and accurate diagnosis of active disease, in which laboratories play a key role, is a prerequisite for any successful TB control programme.^3^,^4^ Over the last 20 years, TB laboratory diagnosis has evolved globally, especially in high-income settings, from being predominantly microscopy- and culture-based to almost universal use of molecular technologies that enable rapid and reliable detection of TB and drug resistance, transmission studies and outbreak tracing.^5–7^ Extensive roll-out of various molecular-based modalities, including line-probe assays (LPAs), genotyping technologies and real-time polymerase chain reaction (PCR) based systems poses specific challenges for diagnostic laboratories. Lack of training, expertise and human resources have been reported in many settings to be major obstacles to the performance of TB laboratory activities.^8^ Although molecular techniques are used widely, only a few published studies have focused on labour costs and, specifically, on the working times necessary for assay execution (predominantly Xpert^®^ MTB/RIF, Cepheid, Sunnyvale, CA, USA).^9–11^ Comprehensive data on hands-on time spent on specific assays is scarce,^9^,^12^ making an accurate calculation of workload in a diagnostic laboratory a challenging task. Correct estimation of workload in a TB diagnostic laboratory is critical for its sustainable management.

To collect workload information, several approaches have been described, including self-reporting, work sampling (collection of data at particular time intervals), time-and-motion (T&M) or questionnaires.^12–14^ Among these, T&M, which requires continuous and independent observation, has been in use since the mid-1940s and is generally considered to be one of the most reliable methods compared with other approaches.^12^,^15^,^16^ T&M is based on splitting procedures into individual steps and recording the time needed to perform the step by independent observers to minimise bias and ensure objectivity and data portability between sites. T&M has proved effective in TB laboratories, as demonstrated in a recent study on a limited range of laboratory assays.^12^

In the present study, we report on the further development of a T&M data acquisition tool and its assessment in six EU reference TB laboratories within the European Reference Laboratory Network for Tuberculosis (ERLTB-Net).

The ultimate aim of the present study was the development of an objective means of recording the actual time spent on running and reporting laboratory assays which could be used nationally and internationally to help in the determination of laboratory workloads, make improvements and justify the use of resources.

## MATERIALS AND METHODS

### Further development of the time-and-motion tool and hands-on time recording

In the current study, we further developed a T&M tool for recording hands-on time for four TB laboratory diagnostic assays. The hands-on time in our study was defined as a time of continuous activity of a staff member (including waiting times of no longer than 15 min) needed to perform the individual steps of an assay. Standard operating procedures (SOPs) for each test, including running the assays, data analysis (where applicable) and reporting, were divided into tasks (please contact corresponding author for details) (Appendix).^*^ Theoretical times per specimen (TTPS) were calculated by dividing hands-on time by the number of specimens in the batch.

At each participating laboratory, bench-active staff members carrying out the tests were continuously followed by an independent observer recording the start and end times of each task. To minimise inter-observer bias, different staff members were observed for each test. The number of specimens processed during each observation and numbers of staff members were recorded (Appendix Table A). Times for preparation, cleaning of the work area and completion of administrative work were included in the calculation (unless stated otherwise). Basic training was provided to observers remotely by providing Excel spreadsheets for time recordings and a one-off teleconference.

### Study design

The study was conducted in six European TB National Reference Laboratories (NRLs) (Table). Laboratories A, C, D and E were located in low TB incidence countries (incidence < 10/100 000), while laboratories B and F were located in medium TB incidence countries (>50/100 000). Timings for each analysed test were recorded in at least two laboratories that had been performing the particular test routinely for a minimum of 1 year.

**Table:**
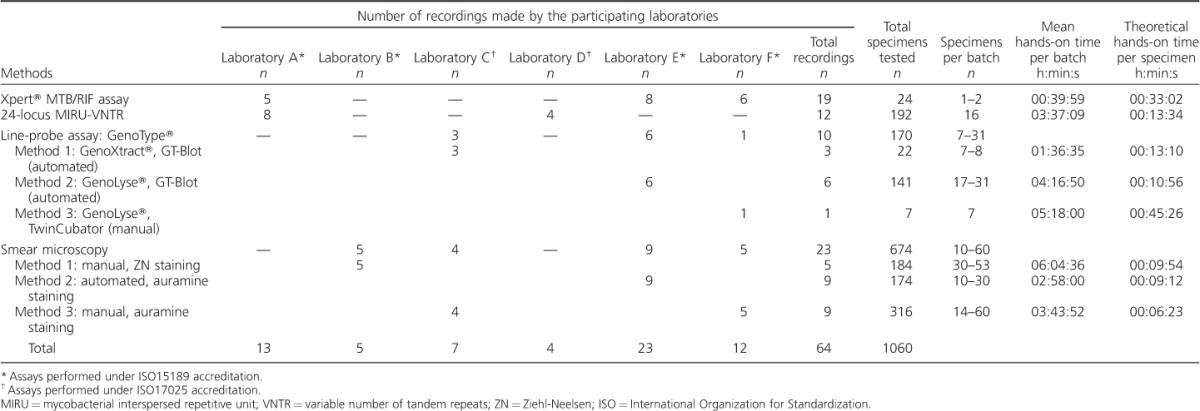
Number of recordings and specimens analysed by each participating National Reference Laboratory and the mean hands-on times and theoretical times per specimen calculated for the various laboratory assays and methodologies

As the study did not involve human subjects and no patient information was assessed or recorded, ethics permission was not sought.

### Laboratory assays

To cover both the diagnostic and reference aspects of TB laboratory activities, we included smear microscopy, Mycobacterium tuberculosis complex (MTC) genotyping using the 24-locus mycobacterial interspersed repetitive unit-variable number of tandem repeats (MIRU-VNTR) technique and detection of MTC and mutations conferring rifampicin resistance using Xpert and LPAs (GenoType^®^, Hain Lifescience, Nehren, Germany, including MTC, common mycobacteria, additional species [CM/AS] assay and MTBDR*plus* assays). Microscopy slides were read according to current World Health Organization standards.^17^,^18^

All tests have been extensively validated,^18^ and are commonly used in TB diagnostic laboratories worldwide. All molecular assays were performed as recommended by their respective manufacturers. Details on the assays performed by individual laboratories and the steps included are given in the Appendix.

### Data analysis

Data were entered into a Microsoft Excel file; the total hands-on time of a test was calculated as the sum of the working times for each task. Except for some Xpert analyses, samples were processed in batches. The theoretical time to process one sample was calculated by dividing the recorded time by the number of samples,^12^ and did not reflect the actual time necessary for the individual processing of a sample.

Correlations, mean times and standard deviations (SDs) (reported as the mean ± SD) were calculated using Microsoft Excel; *P* values were calculated using unpaired *t*-test (GraphPad Prism, San Diego, CA, USA).

## RESULTS

A total of 1060 specimens, including primary (sputum) and reference (M. tuberculosis cultures and crude DNA extracts) samples, were analysed. The number of specimens per batch varied from one (Xpert) to 60 (smear microscopy), with a total of 64 recordings performed.

### Principal findings by assay

**Figure 1:**
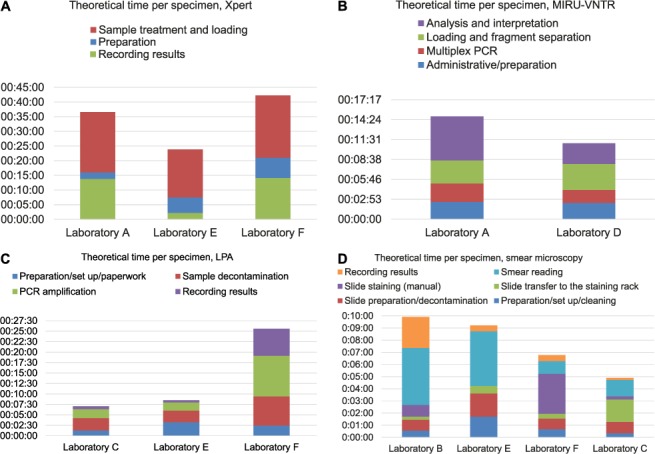
Theoretical processing times per specimen. A) Xpert^®^ MTB/RIF; B) MIRU-VNTR genotyping; C) line-probe assays; D) smear microscopy. MIRU = mycobacterial interspersed repetitive unit; VNTR = variable number of tandem repeats; LPA = line-probe assay; PCR = polymerase chain reaction. This image can be viewed online in colour at http://www.ingentaconnect.com/content/iuatld/ijtld/2018/00000022/00000004/art00017

The mean hands-on times and theoretical times per specimen calculated for the different analysed tests are shown in the Table. The contribution of individual steps into specimen processing times in the different laboratories is presented in Figure 1.

### Xpert^®^ MTB/RIF assay

In total, the assay was observed 19 times in three laboratories (Table) with 23 primary sputum specimens. The mean TTPS in h:min:s was 00:33:02 ± 00:12:32.

Times for specimen preparation, treatment and loading did not vary significantly across the participating laboratories. Variations in working times observed were predominantly linked to differences in the times required for recording and reporting results (e.g., Laboratory A 00:13:48 ± 00:01:06, 95% confidence interval [CI] 00:12:26–00:15:10 vs. Laboratory E 00:02:12 ± 00:00:53, 95%CI 00:01:31–00:02:52, *P* < 0.0001; Figure 1A). Specifically, protocols for reporting results in Laboratory E included only entering results into the Laboratory Information Management System (LIMS), while Laboratories A and F followed more complex multistep procedures, including generation of reports using Xpert software, saving it in a secure location, entering results into the LIMS and reporting validation by a senior staff member.

### Mycobacterial interspersed repetitive unit-variable number of tandem repeats genotyping assay

In participating Laboratories A and D, the time needed to perform 24-locus MIRU-VNTR typing on 16 samples (1 plate) was recorded. The assay was observed 12 times (Table). The mean theoretical hands-on time to analyse one plate was 03:37:09 ± 00:32:22, with a TTPS of 00:13:34 (Table). Time to perform cluster analysis was not included as it was not performed routinely in either participating laboratories.

Similar to the Xpert assay, total TTPS and steps within the procedure did not differ significantly, apart from the times spent on analysis and interpretation (00:06:12 ± 00:00:19, 95%CI 00:05:56–00:06:17 vs. 00:02:58 ± 00:00:33, 95%CI 00:02:05–00:03:50, *P* < 0.0001; Figure 1B). This could be explained mainly by variations in the software packages used (GeneMapper) for the analysis and interpretation of results.

### Line-probe assays

LPAs were observed 10 times in three laboratories. As methods for DNA extraction and hybridisation varied across laboratories, results were analysed separately (Table). The mean TTPS using the GT-Blot machine automated method varied between 10 and 13 min compared with 45 min when the low-throughput manual method (TwinCubator) was used (Table).

Hands-on time and its distribution by steps did not vary significantly between Laboratories C and E. In Laboratory F, sample preparation, PCR, hybridisation and the recording of results took significantly longer, which could be explained in part by the differences in reporting procedures (also noted for Xpert, please see above), as well as significant differences in cleaning and biosafety procedures.

### Smear microscopy

In total, smear microscopy was observed 24 times in four laboratories using three techniques: manual auramine staining (two laboratories), manual Ziehl-Neelsen (ZN) staining (one laboratory) and automated auramine staining (one laboratory, using the Varistain V24-4 Automatic Slide Stainer; Thermo-Scientific, Waltham, MA, USA). Results were recorded and analysed separately for the three methods. The mean theoretical hands-on time per ZN smear using the manual method was 00:09:54 ± 00:00:53, compared with 00:06:23 ± 00:01:36 and 00:09:12 ± 00:01:18 for auramine staining using manual and automatic methods, respectively.

Reading smears took significantly longer for ZN staining than manual auramine staining (00:04:41 ± 00:00:23, 95%CI 00:04:16–00:05:05 vs. 00:01:03 ± 00:00:20, 95%CI 00:00:34–00:01:31, *P* < 0.001); however, reading auramine-stained smears using an automated method took almost as long as ZN (Figure 1D). Overall, despite using an automated auramine staining technique, TTPS in Laboratory E was only marginally shorter than manual ZN staining (Laboratory B), and significantly longer than in Laboratories F and C, which could be explained in part by the more scrupulous procedures needed for slide preparation, decontamination, assay setup and cleaning for automated staining.

### Variation in hands-on times depending on the number of specimens

**Figure 2:**
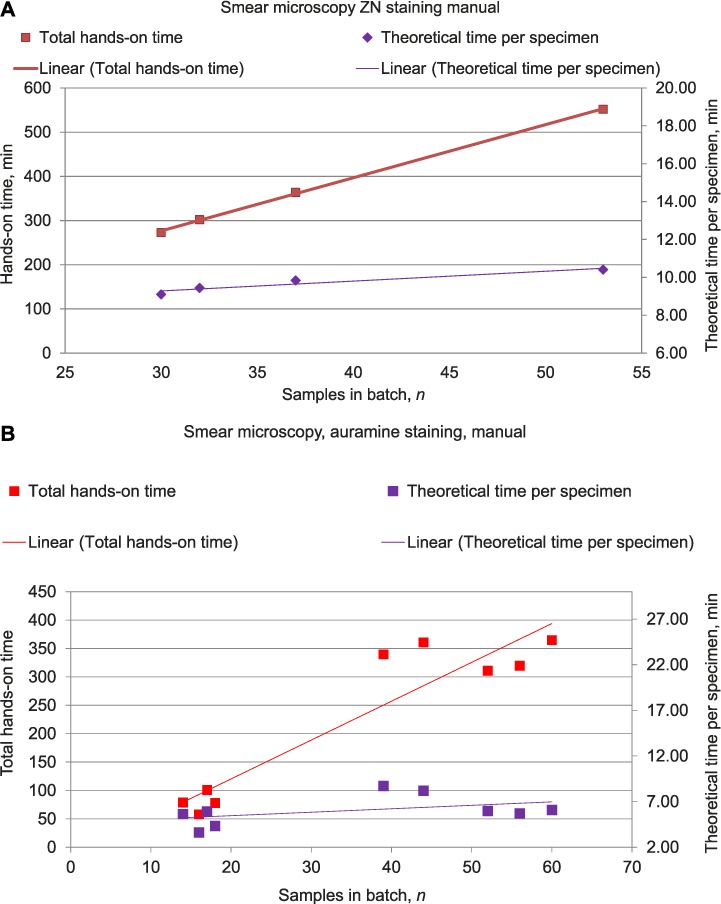
Total hands-on time and theoretical times per specimen for smear microscopy (manual staining methods only). A) ZN staining, manual method; B) Auramine staining, manual method. LPA = line-probe assay; ZN = Ziehl-Neelsen. This image can be viewed online in colour at http://www.ingentaconnect.com/content/iuatld/ijtld/2018/00000022/00000004/art00017

**Figure 3:**
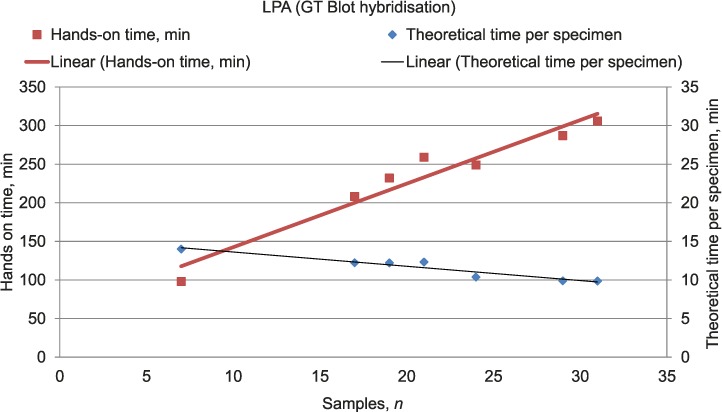
Total hands-on time and theoretical times per specimen for line-probe assays (automated hybridisation using GT-Blot). This image can be viewed online in colour at http://www.ingentaconnect.com/content/iuatld/ijtld/2018/00000022/00000004/art00017

Variations in total hands-on times and TTPS depending on the number of specimens in a batch were calculated for LPA (automated hybridisation system, Laboratories C and E) and smear microscopy (both manual ZN and auramine staining, Laboratories B, E and F) (Figures 2 and 3). Due to a small or constant number of specimens in batches, it was not possible to perform this assessment for Xpert assays, MIRU-VNTR genotyping and other assays performed by individual laboratories only.

Strong positive correlations between the number of specimens per batch and total hands-on times were observed for LPAs and manual microscopic assays (*R* = 0.97, 0.99, and 0.93 respectively), which suggests that the total time needed for the completion of these assays depended heavily on the number of specimens per batch. TTPS using LPA assays negatively correlated with batch size (*R* −0.96), indicating that in larger batches the time spent on individual specimens was shorter (Figure 3). No consistent correlations were seen between batch size and TTPS in microscopic assays (Figure 2).

## DISCUSSION

Optimal allocation of human resources to ensure provision of sustainable high-quality laboratory services is one of the most challenging managerial tasks. Laboratory managers need objective information to make informed decisions while reviewing laboratory activities and considering restructuring or implementation of new techniques. Objective assessment of the human resources needed to perform certain activities is a critical part of any cost-effectiveness analysis. It allows accurate determination of labour and other associated costs, reduction in turnaround times (TAT) and development of an adequate pricing strategy. This could ultimately help the laboratory to stay competitive in the laboratory service market, both nationally and internationally. The lack of data currently available on this topic motivated the current study, which aimed to further develop and validate a universal methodology for accurate determination of the hands-on time necessary for running diagnostic assays performed in TB diagnostic laboratories.

The results of our study confirmed that splitting procedures into steps allowed for a direct comparison of times spent on laboratory assays between laboratories and, more importantly, identification of reasons for the delays and areas for improvement. There were no significant differences in hands-on time spent on pre- and analytical laboratory-based stages of highly standardised and automated methodologies such as Xpert and MIRU-VNTR typing between different laboratories. Times for the Xpert assay (00:39:59 and 00:33:02 per batch and specimen, respectively) were also comparable with earlier estimates.^12^ These findings demonstrate the validity of the T&M methodology and its potential for wider use in diagnostic laboratories in various settings. Times spent on predominantly office-based procedures (recording, reporting and interpretation) were different across participating sites, mainly due to differences in local SOPs and variations in data processing and reporting requirements.

To note, as demonstrated for MIRU-VNTR genotyping procedures, the T&M methodology also allowed identification of areas for potential technical improvements and the need for modernisation. An in-depth analysis of differences in times demonstrated that, in Laboratory A, software was calibrated in a slightly different/suboptimal way, leading to a longer time needed for analysis and VNTR allelic variant assignation. Comparison of the times needed to perform LPAs between laboratories using automated and manual hybridisation techniques clearly showed the role of automation in reducing hands-on time, giving yet another example of how areas for improvement and streamlining could be identified using the T&M tool. However, the T&M model may prove less useful for assays with a greater involvement of manual and/or less standardised work that may be heavily dependent on operator experience (e.g., smear microscopy).

There have been conflicting views on the role of batch processing in reducing the TAT in processing laboratory specimens.^19^,^20^ Analysis of the correlations performed in our study demonstrated that processing specimens in batches can help save time in highly automated assays such as LPAs using robotic devices (TTPS 00:14:00 vs. 00:09:45 for batches comprising 7 and 31 specimens, respectively). Batching specimens for methodologies with greater manual work involvement is less effective.

We believe that the working times reported in our study are generalisable and can contribute to workload estimates in other diagnostic and reference TB laboratories; generic templates (provided on request) could be modified to suit the laboratory staffing levels, SOPs and laboratory assays used. Laboratory accreditation is important, as it ensures strict adherence to SOPs, therefore minimising the potential bias related to staffing levels and other operational issues.

Availability of a tool for objective time recording is especially important for continuity arrangements in case of emergency and/or outsourcing specific activities to other laboratories to ensure an optimal (or at least manageable) work distribution that does not exceed existing capacity. Our data could also be used to compare current techniques and, eventually, to support technical change in other laboratories.

Although recordings were performed by junior and/or new staff members to minimise potential bias, changes in the behaviour of staff members as a consequence of being observed cannot be excluded, and could be considered one of the study limitations. Additional study limitations included the relatively small number of recordings; intra- and inter-observer variability could therefore not be assessed. One strength of our study was that participating laboratories were located in both low and medium TB settings.

We concluded that hands-on time recording based on T&M principles can be considered a universal and objective methodology contributing to workload assessment in TB diagnostic laboratories. Comparison of workload between laboratories will ensure fairer distribution of work in the future, and also help laboratory managers justify their personnel needs when implementing novel, time-saving, cost-effective technologies while also identifying areas for improvement. Our study also demonstrated the value of networking activities in sharing expertise and developing methodologies that could be used to improve quality and laboratory performance within ERLTB-Net and beyond.

## Figures and Tables

**Table A:**
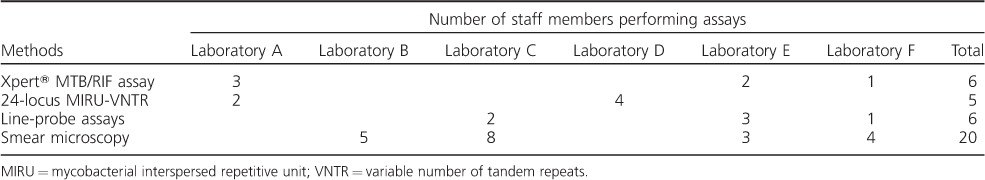
Number of staff members performing laboratory assays in Laboratories A–F

## Footnotes

^*^ The appendix is available in the online version of this article, at http://www.ingentaconnect.com/content/iuatld/ijtld/2018/00000022/00000004/art00017

## APPENDIX

### TIME AND MOTION STUDY METHODOLOGY: BRIEF NOTES

#### Time and motion: general recommendations

Please use the Excel^™^ file (provided on request) and select the methodology you are interested in.

Spreadsheets contain formulae, so please be careful when entering data and making changes to preserve the integrity and functionality of the programme.

Tasks/steps within Excel spreadsheets could be modified based on standard operating procedures (SOPs) used in the laboratory.

Recordings should ideally be performed by a junior/new/external staff member to minimise bias.

#### Critical points

- All procedures should be split into tasks according to existing SOPs
- Strict adherence to SOP is critical.

#### Recommendations on how to record times and enter recordings into the Excel spreadsheets

- Strict adherence to SOP is critical. Please use a wall clock or any timer to record the start time and endtime of each step. Timings should be rounded to the nearest minute unless the timing is very short, in which case it is to be rounded to the nearest 10 s.
- Please enter the following in the Excel spreadsheet:
  ○ Date of the recording
  ○ Name of the staff member running an assay
  ○ Number of specimens per batch
  ○ Start and end times for each step
  ○ The time taken for each step, total working time and theoretical time per specimen (TTPS) will be calculated automatically.
- Tasks to be recorded:
  ○ Paperwork
  ○ Assay set up
  ○ Assay running
  ○ Cleaning up
  ○ Interpretation and reporting should be recorded
  ○ Waiting times up to 15 min should be included in relevant steps
- Tasks not to be recorded:
  ○ Opening of post (receipt of samples).
